# *Ex vivo* expansion of human T cells for adoptive immunotherapy using the novel Xeno-free CTS Immune Cell Serum Replacement

**DOI:** 10.1038/cti.2014.31

**Published:** 2015-01-16

**Authors:** Corey Smith, Grethe Økern, Sweera Rehan, Leone Beagley, Sau K Lee, Tanja Aarvak, Karoline W Schjetne, Rajiv Khanna

**Affiliations:** 1 QIMR Centre for Immunotherapy and Vaccine Development and Tumour Immunology Laboratory, Department of Immunology, QIMR Berghofer Medical Research Institute, Brisbane, Queensland, Australia; 2 Thermo Fisher Scientific, Oslo, Norway

## Abstract

The manufacture of clinical grade cellular products for adoptive immunotherapy requires *ex vivo* culture and expansion of human T cells. One of the key components in manufacturing of T cell therapies is human serum (HS) or fetal bovine serum (FBS), which can potentially expose immunotherapy recipient to adventitious infectious pathogens and are thus considered as non-cGMP compliant for adoptive therapy. Here we describe a novel xeno-free serum replacement (SR) with defined components that can be reproducibly used for the production of clinical grade T-cell therapies in combination with several different cell culture media. Dynabeads CD3/CD28 Cell Therapy System (CTS)-activated or antigen-specific T cells expanded using the xeno-free SR, CTS Immune Cell SR, showed comparable growth kinetics observed with cell culture media supplemented with HS or FBS. Importantly the xeno-free SR supplemented medium supported the optimal expansion of T cells specific for subdominant tumour-associated antigens and promoted expansion of T cells with central memory T-cell phenotype, which is favourable for *in vivo* survival and persistence following adoptive transfer. Furthermore, T cells expanded using xeno-free SR medium were highly amenable to lentivirus-mediated gene transduction for potential application for gene-modified T cells. Taken together, the CTS Immune Cell SR provides a novel platform strategy for the manufacture of clinical grade adoptive cellular therapies.

Adoptive immunotherapy with *ex vivo*-derived T cells is emerging as a powerful tool to treat disease in a range of clinical settings.^1, 2^ The primary clinical settings targeted for adoptive immunotherapy over the past two decades have been viral diseases associated with immune-compromise^3, 4^ and cancer,^2, 5^ which both have shown great success. In these settings, the *in vitro* expansion of T-cell populations from either peripheral blood mononuclear cells (PBMC), gene-modified T cells or tumour infiltrating lymphocytes has been used to improve the functional properties of both CD8^+^ cytotoxic T lymphocytes and CD4^+^ T cells prior to infusion. More recent studies have begun to employ the adoptive transfer of T cells encoding recombinant receptors, typically via delivery with a viral vector system, to improve recognition of tumour cells. This has included the introduction of recombinant T-cell receptors that target defined tumour-associated peptide epitopes in complex with major histocompatibility molecules,^6^ and chimeric antigen receptors that contain antibody chains targeting molecules expressed on the surface of tumour cells.^5, 7^ Recent observations have shown the great potential of using such approaches in the treatment of malignant disease. Other recent approaches have begun to employ *ex vivo* culture to generate regulatory T cells for the treatment of autoimmune disease or graft-versus-host disease,^8, 9, 10^ further emphasising the potential of *ex vivo* expanded T cells for targeted treatment of many human diseases.

Serum supplementation, traditionally with fetal bovine serum (FBS) or human serum (HS), has been a mainstay for *in vitro* tissue culture of mammalian cells, providing essential factors required for survival and growth of cells. The manufacture of T cells for adoptive therapy is also dependent upon the provision of a serum supplement, either FBS or HS, to optimise the generation and function of *ex vivo* expanded T cells. While improved tissue culture media formulations have been developed that provide some incremental improvements in T-cell growth *ex vivo*, these still provide inferior growth kinetics to serum-supplemented media,^11^ particularly in settings where expansion is not induced by non-specific polyclonal activation. However, given increasing regulatory requirements associated with the use of serum in the manufacture of cells for adoptive therapy, there is a pressing need for the development of alternative approaches to the use of both animal-derived and HS.^11^ In this study we have investigated the use of a xeno-free serum replacement (SR), Cell Therapy System (CTS) Immune Cell SR, as an alternative to both HS and FBS. We demonstrate, using clinically relevant expansion protocols, that CTS Immune Cell SR promotes the efficient *ex vivo* expansion of polyclonally activated T cells, with yields similar to that generated in HS. We also show that CTS Immune Cell SR can substitute for the use of FBS in the expansion of T cells specific for two clinically important human herpesviruses, Epstein Barr Virus (EBV) and human Cytomegalovirus (CMV), and demonstrate that culture in CTS Immune Cell SR can enhance the generation of subdominant T-cell responses specific for tumour-associated antigens.

## Results

### CTS Immune Cell SR supports *ex vivo* expansion of polyclonal activated T cells

**Figure 1:**
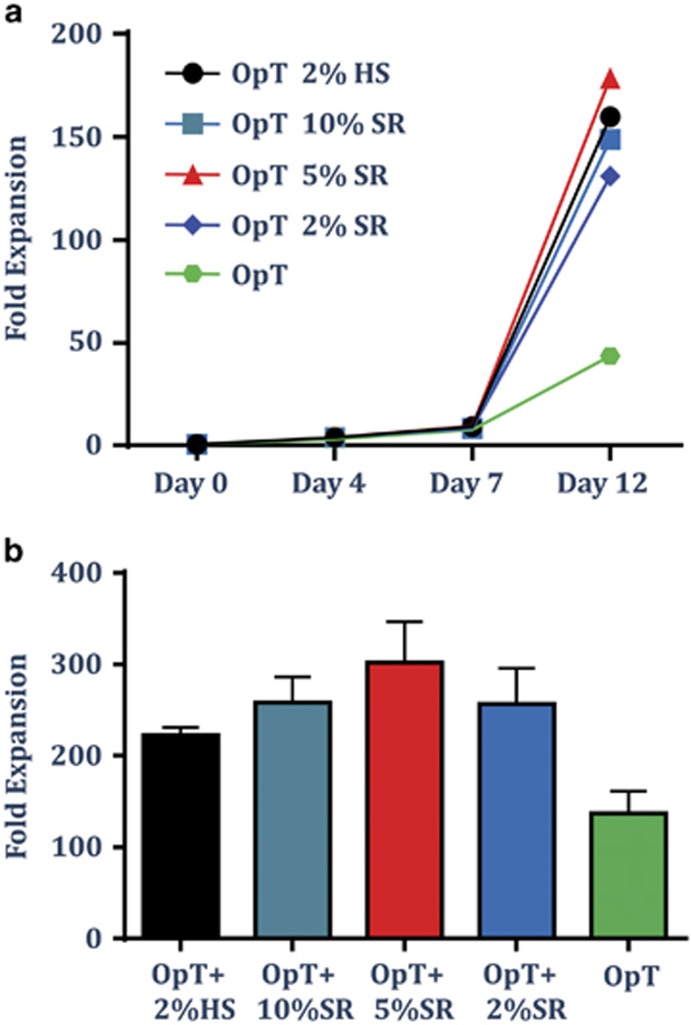
CTS Immune Cell SR supports the *ex vivo* expansion of polyclonal activated T cells. T cells from PBMC were isolated and activated using CTS Dynabeads CD3/CD28 and cultured in OpTmizer cell culture medium supplemented with pooled human AB serum (HS 2%), titrated amounts of CTS Immune Cell SR (2–10%) or no serum. Cells were fed every 1–2 days. (**a**) Analysis of the growth kinetics from one representative donor. (**b**) Data represent the average±s.d. of fold expansion of T cells at the end of culture (day 12) from four donors.

*Ex vivo* expansion of T cells activated with CTS Dynabeads CD3/CD28 is a commonly used protocol for production of T cell products for cell therapy.^12, 13, 14^ Current protocols employ several different cell culture media, all of which are supplemented with pooled human AB serum to increase total fold expansion of T cells. To test whether T cells can expand to the same extent using a xeno-free chemically defined SR, polyclonal T cells from healthy blood donors were activated using Dynabeads and growth kinetics was monitored for a 2-week period. Cells were cultured in CTS OpTmizer T-cell Expansion SFM (Life Technologies, Carlsbad, CA, USA) supplemented with 2% HS or titrated amounts of CTS Immune Cell SR, at a range of 0, 2, 5 or 10%. Cells were fed every 1–2 days and counted at day 4, 7 and 12 (Figure 1). OpTmizer cell culture media supplemented with HS or SR showed similar growth kinetics as shown by one representative donor (Figure 1a) or total fold expansion at the end of the culture as shown by an average of four donors (Figure 1b).

**Figure 2:**
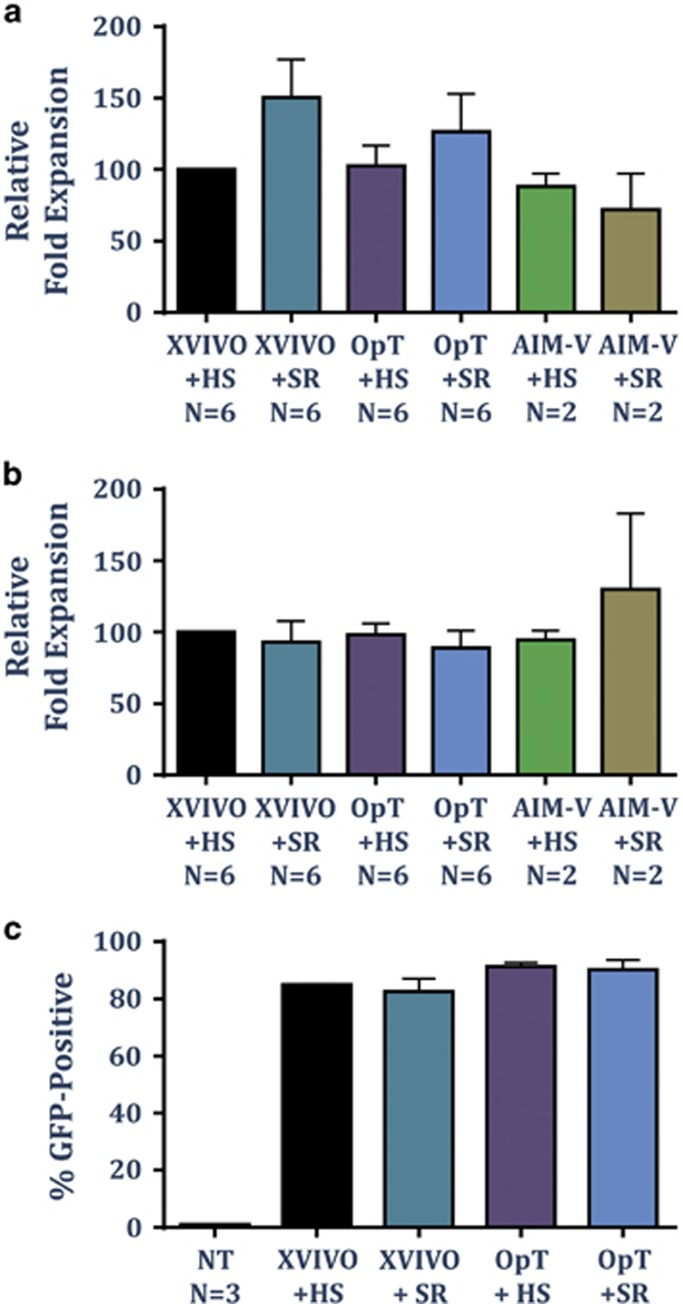
CTS Immune Cell SR supports CD8+ and CD4+ T-cell expansion using the rapid expansion protocol. T cells were activated using OKT-3, IL-2 (3000 U ml^−1^) and irradiated allogenic feeder cells (1:100) in different cell culture media supplemented with either HS or 10% SR, and supplemented with fresh media every 1–2 day. On day 13, cells were counted and stained for CD4 and CD8. Data represent the average±s.d. relative fold expansion of CD8^+^ T cells (**a**) or CD4^+^ T cells (**b**) when compared with cells cultured in XVIVO+HS. (**c**) T cells from PBMC were isolated and activated using CTS Dynabeads CD3/CD28 and cultured in XVIVO15 or OpTmizer cell culture medium supplemented with HS (5% HS with XVIVO15 and 2% HS with OpTmizer) or 10% SR. After 16–20 h, cells were transduced with pELNS-GFP lentiviral vector. At day 7, GFP expression was analysed using a flow cytometer. Data represent average±s.d. of the percentage of GFP^+^ cells from a total of 3 donors. NT, non-transduced cells.

The rapid expansion protocol, first described by the Rosenberg group, uses anti-CD3 monoclonal antibody (OKT3), high dose interleukin-2 (IL-2) and irradiated allogenic feeder cells to generate T cell for adoptive therapy from tumour infiltrating lymphocytes.^15^ To study whether SR could support expansion of T cells activated using soluble anti-CD3 monoclonal antibody and feeder cells, polyclonal T cells were activated according to the rapid expansion protocol and cultured in XVIVO15, OpTmizer or CTS AIM-V supplemented with either HS or SR. On day 12, total fold expansion of CD4^+^ and CD8^+^ T cells was analysed by flow cytometry. A similar fold expansion in both CD4^+^ and CD8^+^ T cells was evident in all culture media tested (Figures 2a and b). Furthermore the expansion of both T cell subsets was maintained when HS was replaced with the xeno-free chemically defined SR regardless of the cell culture media used.

Polyclonal activation of T cells is commonly used in the generation of gene-modified T cells encoding either tumour-specific chimeric antigen receptors or T-cell receptors.^6, 13, 14, 16^ To demonstrate the capacity of SR to support the *ex vivo* expansion of gene-modified T cells, polyclonal T cells activated with CTS Dynabeads CD3/CD28 were transduced with a GFP-expressing lentiviral vector (16–20 h after activation) and the expression of GFP was analysed by flow cytometry on day 7. As a control, T cells from the same donors were expanded in the corresponding cell culture media supplemented with HS. A high transduction efficiency was evident in both XVIVO15 and OpTmizer supplemented with either HS or SR (Figure 2c), demonstrating the capacity of CTS Immune Cell SR to support the growth of gene-modified T cells.

### CTS Immune Cell SR supports the expansion of virus-specific T cells

**Figure 3:**
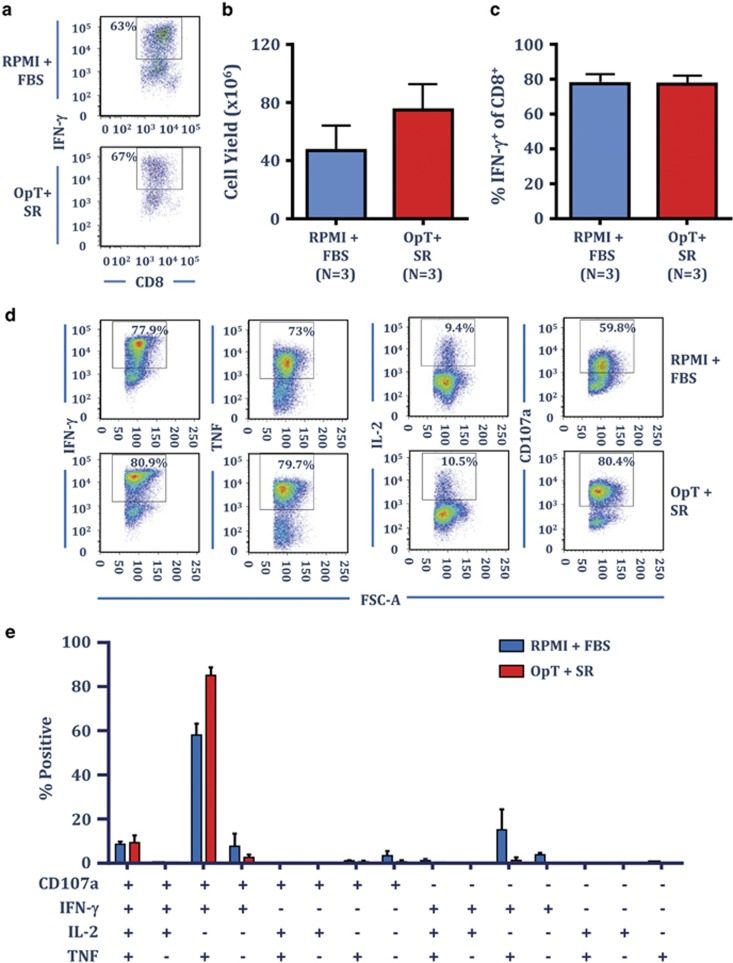
CTS Immune Cell SR supports the *ex vivo* expansion of CMV-specific T cells. PBMC from healthy CMV-seropositive donors were cultured with autologous PBMC pulsed with a pool of CMV-encoded CD8^+^ T-cell peptide epitopes in either RPMI–FBS or OpTmizer-SR. T-cell cultures were supplemented with 50% fresh media containing 120 IU ml^−1^ IL-2 after 3 days and every 3–4 days after. On day 14, cell numbers were determined using trypan blue exclusion, then T-cell specificity was determined using an intracellular IFN-γ assay following recall with a pool of defined CMV-encoded, CD8^+^ T-cell peptide epitopes. (**a**) Representative analysis from the same donor stimulated with the CMV peptide pool and cultured in either RPMI–FBS or OpTmizer-SR is shown. (**b**) Data represents the mean±s.e.m. from three donors of the number of viable cells following CMV peptide pool stimulation in RPMI–FBS or OpTmizer-SR. (**c**) Data represent the mean±s.e.m. from three donors of the frequency of CMV-specific CD8^+^ T cells following CMV peptide pool stimulation in RPMI–FBS or OpTmizer-SR. (**d**) CMV peptide pool expanded T cells were assessed for multiple cytokine production (IFN-γ, TNF and IL-2) and degranulation (CD107a) following recall with cognate peptides. Representative dot plots are shown from the same donor cultured in either RPMI–FBS or OpTmizer-SR. (**e**) Data represent the mean±s.e.m. from three donors of the proportion of CMV-specific CD8^+^ T cells producing different combinations of IFN-γ, TNF, IL-2 and CD107a in RPMI–FBS or OpTmizer-SR.

To evaluate the efficacy of OpTmizer and CTS Immune Cell SR in promoting the expansion of viral antigen-specific T cells, we investigated their use in the production of CMV-specific T cells using a peptide-based stimulation protocol we have successfully employed to generate cellular therapies for CMV-associated disease in transplant patients and to treat CMV-associated glioblastoma multiforme.^17, 18^ In this setting, PBMC from three CMV-seropositive individuals were exposed to a pool of custom designed CMV-specific T-cell peptide epitopes, then cultured for 2 weeks in either RPMI supplemented with 10% FBS or OpTmizer supplemented with 5% SR. All cultures were supplemented with 50% fresh culture medium containing 120 IU ml^−1^ recombinant IL-2 on day 3 and every 3 days thereafter. To assess the impact of OpTmizer supplemented with SR on T-cell expansion, at the completion of the culture period, T-cell numbers were enumerated using trypan blue exclusion and T-cell specificity was assessed using a standard intracellular interferon-γ (IFN-γ) assay. Representative intracellular IFN-γ analysis from a single donor comparing RPMI–FBS and OpTmizer-SR is shown in Figure 3a. Consistent with the results following polyclonal stimulation, cell yield was comparable or increased following culture in the presence of OpTmizer-SR (Figure 3b), and the frequency of IFN-γ producing CMV-specific T cells was similar (Figure 3c).

The polyfunctional profile of T lymphocytes, including the capacity to degranulate (measured by the surface expression of CD107a) and the production of multiple cytokines (IFN-γ, TNF (tumor necrosis factor) and IL-2) is associated with the better control of disease.^19, 20^ To compare the functional properties of T cells expanded in either RPMI–FBS or OpTmizer-SR, we assessed the polyfunctional profile in CMV-specific T cells from the three donors after 14 days in culture. Representative analysis for a single donor stimulated with the CMV peptide pools in either RPMI–FBS or OpTmizer-SR is shown in Figure 3d. In both settings the majority of cells showed a typical effector profile characterised by the production of IFN-γ, TNF and CD107a, and a similar proportion (~10%) of cells also produced IL-2 (Figure 3e).

### CTS immune cell SR provides optimal expansion of T cells specific for subdominant tumour-associated EBV antigens

**Figure 4:**
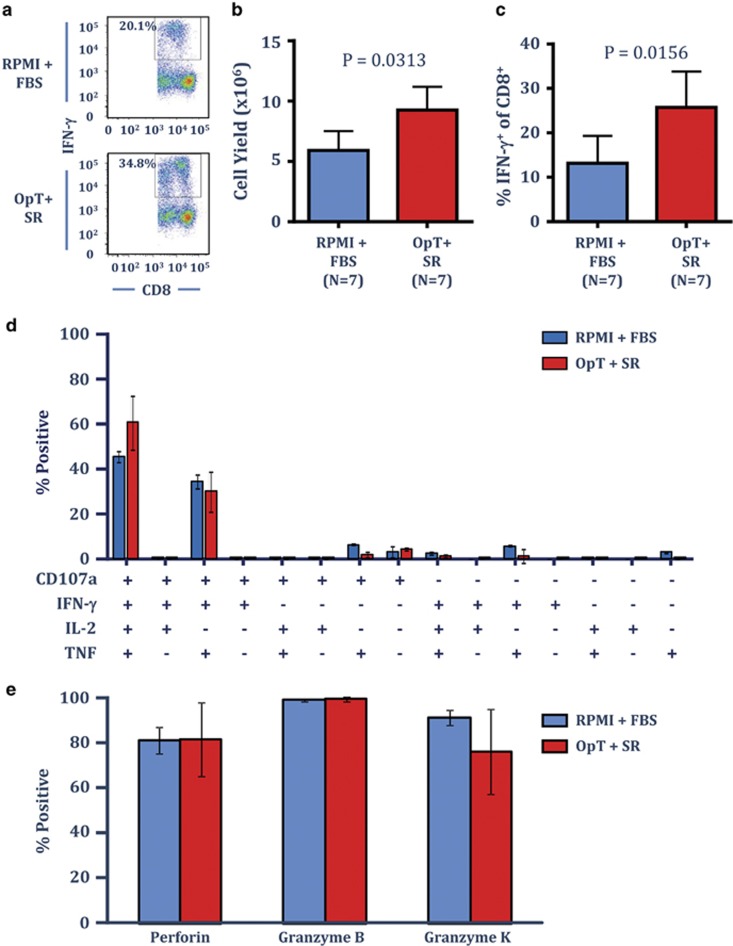
CTS Immune Cell SR optimises the expansion of T cells specific for the subdominant tumour-association EBV antigens, LMP1&2 and EBNA1. PBMC from healthy EBV-seropositive donors were cultured with autologous AdE1-LMPpoly infected PBMC in either RPMI–FBS or OpTmizer supplemented with SR. T-cell cultures were supplemented with 50% fresh media containing 120 IU ml^−1^ IL-2 after 3 days and every 3–4 days after. On day 14, cell numbers were determined using trypan blue exclusion, then T-cell specificity was determined using an intracellular IFN-γ assay following recall with a pool of defined LMP1&2 and EBNA1. (**a**) Representative analysis from the same donor stimulated with AdE1-LMPpoly and cultured in either RPMI–FBS or OpTmizer-SR is shown. (**b**) Data represent the mean±s.e.m. from seven donors of the number of viable cells following AdE1-LMPpoly stimulation in RPMI–FBS or OpTmizer-SR. (**c**) Data represent the mean±s.e.m. from seven donors of the frequency of LMP1&2/EBNA1-specific CD8^+^ T cells following AdE1-LMPpoly stimulation in RPMI–FBS or OpTmizer-SR. (**d**) AdE1-LMPpoly expanded T cells were assessed for multiple cytokine production (IFN-γ, TNF and IL-2) and degranulation (CD107a) following recall with cognate peptides. Data represent the mean±s.e.m. from three donors of the proportion of EBV-specific CD8^+^ T cells producing different combinations of IFN-γ, TNF, IL-2 and CD107a in RPMI–FBS or OpTmizer-SR. (**e**) AdE1-LMPpoly expanded T cells, were stained with the HLA B35/HPVGEADYFEY pentamer, then assessed for the intracellular expression of granzymes B and K and perforin. Data represent the mean±s.e.m. from two donors (use average and s.d.) of the proportion of HPVGEADYFEY-specific CD8^+^ T cells producing each effector molecule in RPMI–FBS or OpTmizer-SR.

EBV LMP1&2- and EBNA1-specific T cells offer therapeutic potential to treat EBV-associated malignancies, including nasopharyngeal carcinoma and EBV-associated lymphomas.^21, 22^ Unlike the immunodominant CMV-specific T cells, EBV LMP1&2- and EBNA1-specific T cells are classically subdominant EBV-specific T cells associated with very low frequencies in peripheral blood and we have previously shown that optimal expansion is achieved using an adenoviral vector encoding the minimal CD8^+^ T cell epitopes (known as AdE1-LMPpoly) and cultured in RPMI–10% FBS.^23^ To determine if OpTmizer supplemented with CTS immune cell SR could provide efficient culture conditions in a clinically compliant protocol used to generate LMP1&2- and EBNA1-specific T cells, PBMC from seven EBV-seropositive individuals were cultured with autologous PBMC infected with the AdE1-LMPpoly vector as outlined previously, either in RPMI–FBS or OpTmizer-SR. All cultures were supplemented with 50% fresh culture medium containing 120 IU ml^−1^ recombinant IL-2 on day 3 and every 3 days thereafter. On day 14, T-cell cultures were assessed for cell number, and the frequency of LMP1&2- and EBNA1-specific T cells using intracellular cytokine analysis. Representative intracellular IFN-γ analysis from a single donor is shown in Figure 4a. OpTmizer supplemented with SR resulted in a significant increase in overall cell yield in all of the donors tested relative to cells cultured in RPMI–FBS (Figure 4b). Similarly, all cultures displayed a significant increase in the frequency of LMP1&2- and EBNA1-specific CD8^+^ T cells relative to RPMI–FBS (Figure 4c). These observations demonstrate that OpTmizer cell culture media with CTS immune cell SR can support the *ex vivo* expansion of T cells specific for subdominant tumour-associated viral antigens, with at least similar efficiency to the RPMI–FBS.

To explore the functional properties of the EBV-specific T cells following *in vitro* stimulation in the presence of OpTmizer-SR, we assessed the polyfunctional profile of the LMP1&2- and EBNA1-specific T cells following expansion in three EBV-seropositive donors; and investigated the expression of the effector molecules granzyme B, granzyme K and perforin in EBV MHC-multimer-specific T cells. Similar to the observations in CMV-specific T cells, the polyfunctional profile of EBV-specific T cells was comparable following culture in either OpTmizer-SR or RPMI–FBS, whereby in both culture conditions the majority of cells were either CD107a^+^IFN-γ^+^TNF^+^IL-2^+^ or CD107a^+^IFN-γ^+^TNF^+^IL-2^−^ (Figure 4d). Similarly, T cells cultured in either OpTmizer-SR or RPMI–FBS displayed a similar effector profile, with the majority of EBV MHC-multimer-specific cells expressing perforin and granzymes B and K (Figure 4e).

### Culture of antigen-specific T cells in OpTmizer medium supplemented with CTS immune cell SR expands T cells with central memory phenotype

**Figure 5:**
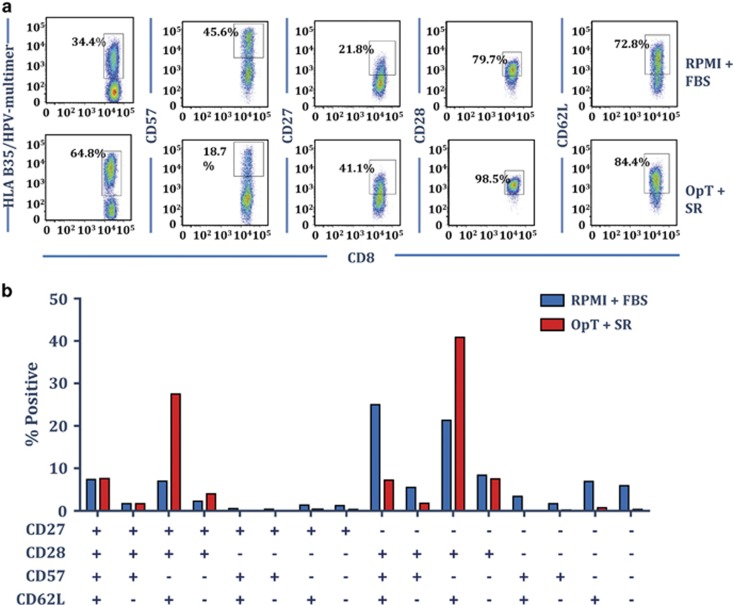
CTS Immune Cell SR promotes a central memory T-cell phenotype in expanded EBV-specific T cells. AdE1-LMPpoly expanded T cells were stained with the HLA B35/HPVGEADYFEY pentamer prior to assessing the surface expression of CD27, CD28, CD57 and CD62L. (**a**) Representative dot plots of the expression of CD27, CD28, CD57 and CD62L by HPVGEADYFEY-specific T cells are shown from the same donor cultured in either RPMI–FBS or OpTmizer-SR. (**b**) Data represent the mean±s.e.m. from three donors of the proportion of HPVGEADYFEY-specific CD8^+^ T cells expressing different combinations of CD27, CD28, CD57 and CD62L in RPMI–FBS or OpTmizer-SR.

To explore the impact of culturing virus-specific T cells in OpTmizer supplemented with CTS immune cell SR on the immunophenotype of expanded T cells, EBV MHC-multimer-specific T cells were assessed for the surface expression of CD27, CD28, CD57 and CD62L. Representative analysis of MHC-multimer staining and surface marker expression from one donor is shown in Figure 5a. Interestingly, while culture in OpTmizer-SR increased the frequency of MHC-multimer-specific T cells, it also resulted in a less terminally differentiated phenotype, most evident by a reduction in the proportion of cells expressing the terminal differentiation marker CD57. Although T cells cultured in RPMI–FBS were predominantly CD27−CD28+CD57+CD62L+ or CD27−CD28+CD57−CD62L+, T cells cultured in OpTmizer-SR were predominantly CD27+CD28+CD57−CD62L+ or CD27−CD28+CD57−CD62L+, a typical phenotype of T-central memory cells (Figure 5b). Previous observations have suggested that adoptive therapy with less differentiated central memory T-cell populations can offer increased survival and *in vivo* persistence of T cells following transfer.^24^

### A combination of CTS Immune Cell SR and the G-Rex culture system promotes optimal expansion of antigen-specific T cells

**Figure 6:**
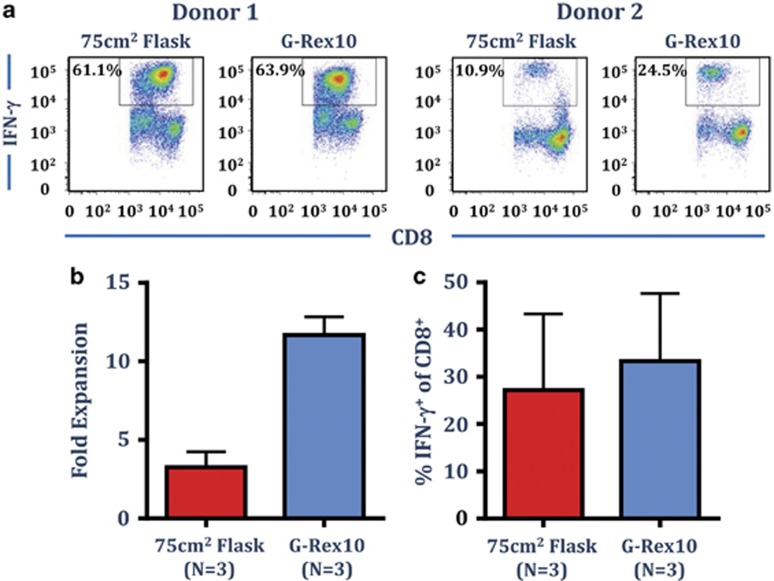
Optimal expansion of EBV LMP1&2- and EBNA1-specific T cells in OpTmizer supplemented with CTS Immune Cell SR in G-Rex10 flasks. PBMC from three EBV-seropositive donors were cultured with autologous AdE1-LMPpoly infected PBMC in OpTmizer-SR (final concentration 5%) in either a 75 cm^2^ flask or a G-Rex10 flask. T cell cultures were supplemented with 50% fresh media containing 120 IU ml^−1^ IL-2 after 3 days and every 3–4 days after. On day 14, cell numbers were determined using trypan blue exclusion, prior to determining T-cell specificity using an intracellular IFN-γ assay following recall with a pool of defined LMP1&2 and EBNA1 CD8^+^ T-cell peptide epitopes. (**a**) Representative intracellular IFN-γ analysis from two different donors is shown. (**b**) Data represent the mean±s.e.m. of the fold expansion of viable cells as determined by trypan blue. (**c**) Data represent the mean±s.e.m. of frequency of LMP1&2/EBNA1-specific CD8^+^ T cells.

The G-Rex culture systems have been designed to support optimal cell growth through improved gas exchange. To investigate the use of the G-Rex culture system with OpTmizer supplemented with SR, PBMC from three EBV-seropositive donors were cultured with autologous AdE1-LMPpoly infected PBMC in OpTmizer-SR, in either a 75 cm^2^ tissue culture flask, or in a G-Rex10 culture vessel. As outlined above, cultures were supplemented with fresh media containing 120 IU ml^−1^ IL-2 on day 3 and every 3–4 days thereafter. Cell yield and the frequency of EBV-specific T cells were assessed after 14 days. Representative intracellular IFN-γ analysis from two donors is shown in Figure 6a. Culture in the G-Rex flasks had a dramatic impact on cell yield; generating a 9- to 13-fold expansion in absolute cell numbers compared with a 2- to 5-fold expansion in the standard 75 cm^2^ tissue culture flask (Figure 6b). Both flasks generated a similar frequency of IFN-γ producing EBV-specific cells (Figure 6c); demonstrating that the CTS OpTmizer T-cell Expansion SFM supplemented with CTS Immune Cell SR can be used effectively to optimise the expansion of antigen-specific T cells in G-Rex culture system.

### IL-2 is sufficient to support the optimal expansion of both CD8^+^ and CD4^+^ T cells in OpTmizer cell culture media supplemented with CTS Immune Cell SR

**Figure 7:**
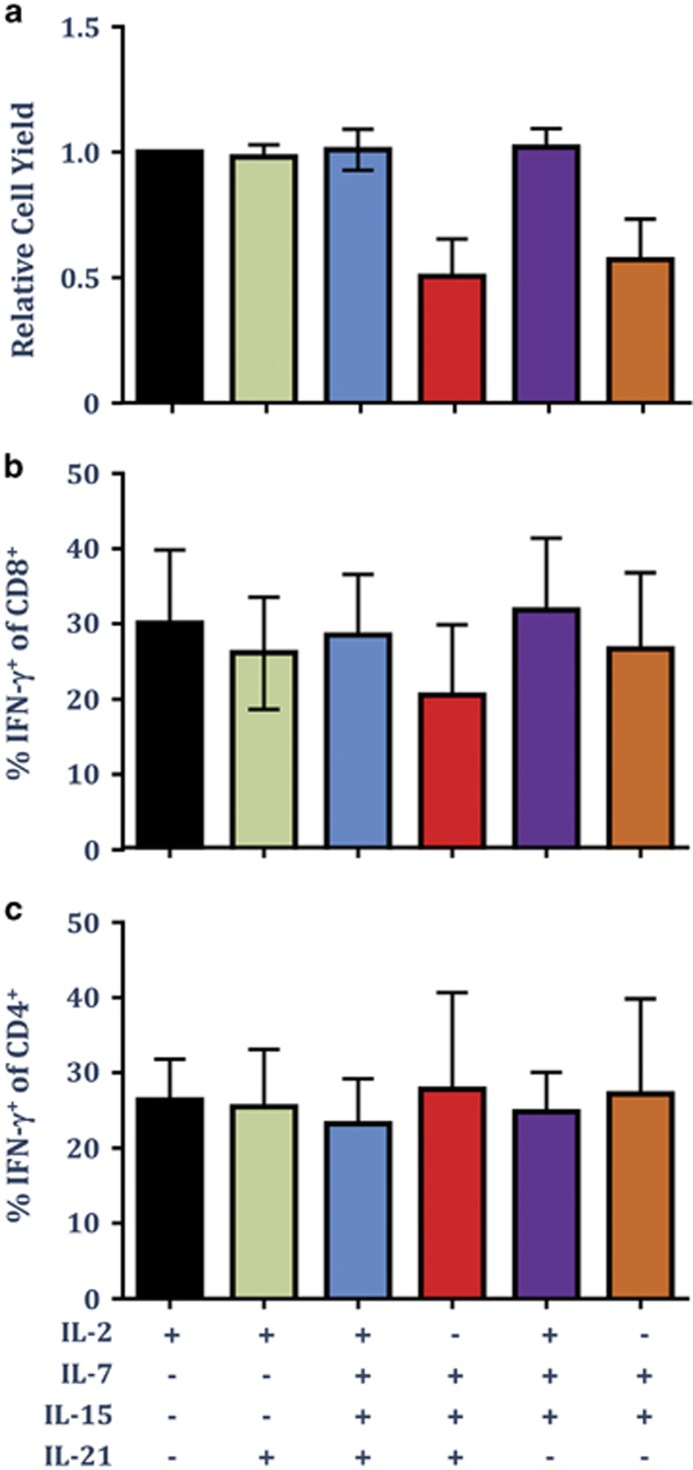
IL-2 is sufficient to support the optimal expansion of both CD8^+^ and CD4^+^ T cells in OpTmizer supplemented with CTS Immune Cell SR. PBMC from five CMV-seropositive donors were cultured with autologous PBMC pulsed with a pool of CMV-encoded CD8^+^ and CD4^+^ T cell peptide epitopes in OpTmizer-SR supplemented with or without 30 ng ml^−1^ IL-21. On day 3 and every 3 days thereafter, cultures were supplemented with IL-2, and/or IL-7 (10 ng ml^−1^) and IL-15 (10 ng ml^−1^). On day 14, cell numbers were determined using trypan blue exclusion, then T-cell specificity was determined using an intracellular IFN-γ assay following recall with the pool of CMV-encoded CD8^+^ and CD4^+^ T-cell peptide epitopes. (**a**) Data represent the mean±s.e.m. of the relative expansion of cells compared with cells cultured in IL-2 alone. (**b**) Data represent the mean±s.e.m. of frequency of CMV-specific CD8^+^ T cells. (**c**) Data represent the mean±s.e.m. of frequency of CMV-specific CD4^+^ T cells.

In addition to IL-2, many studies have investigated the use of different γC-cytokines, including IL-7, IL-15 and IL-21, in promoting optimal expansion of T cells for adoptive therapy.^25, 26, 27^ We therefore next assessed the impact of culture antigen-specific T cells in OpTmizer-SR with IL-7, IL-15 and IL-21, with and without IL-2 following *in vitro* stimulation with the CMV-specific peptide pool. Cultures were supplemented with IL-21 once at the time of stimulation, and with IL-2, IL-7 and IL-15 every 3 days. In addition to CMV-specific CD8^+^ T cells, we also assessed the expansion of CMV-specific CD4^+^ T cells following *in vitro* culture in different combinations of cytokines. Analysis of the relative cell yield following expansion revealed that the optimal expansion of total cells was dependent upon the addition of IL-2 (Figure 7a). Although the addition of IL-21, IL-7 and IL-15 did not enhance the cell yield, culture in IL-7 and IL-15 in the absence of IL-2 reduced the overall cell yield. Additionally, culture in IL-2 alone was sufficient to optimise the expansion of both CMV-specific CD8^+^ T cells (Figure 7b) and CMV-specific CD4^+^ T cells (Figure 7c). These observations confirm efficient expansion of both CD8^+^ and CD4^+^ antigen-specific T cells in OpTmizer cell culture media supplement with CTS Immune Cell SR and demonstrate that the addition of other γC-cytokines does not synergise the expansion of antigen-specific T cells.

## Discussion

Adoptive immunotherapy based on *ex vivo* expanded autologous or antigen-specific T cells has emerged as a powerful tool to treat human cancers and infectious complications.^3, 28, 29^ Over the last decade, the manufacturing process for T-cell therapies has been extensively refined to improve the quality of effector cells and increase the speed of production.^30^ Supplementation of growth medium with HS or FBS is an essential component for manufacturing of cellular immune therapies. However, this process can potentially expose immunotherapy recipient to adventitious infectious pathogens and thus limits clinical application of cellular therapies. Furthermore, infusion of cells expanded using FBS can lead to hypersensitivity reactions due to development of specific antibodies,^31^ although such adverse reactions are rare and predominantly occur following multiple infusions. While Although emergence of serum-free growth medium has provided new platform strategies for safe clinical translation of cellular therapies, these formulations fail to match the efficiency of *ex vivo* T-cell expansion using traditional growth medium supplemented with HS or FBS. In this study we report the development and *ex vivo* assessment of a novel xeno-free chemically defined SR, CTS Immune Cell SR as an alternative to HS or FBS. We demonstrate that CTS Immune Cell SR is highly efficient in expanding polyclonal, antigen-specific and gene-modified human T cells, with yields similar to that generated with cell culture medium supplemented with HS or FBS.

Using both polyclonal stimulation and viral antigen-specific activation, we found that different culture medium formulations (XVIVO, CTS AIM-V and CTS OpTmizer T-cell Expansion SFM) supplemented with CTS Immune Cell SR consistently expanded polyclonal and virus-specific T cells. The expansion kinetics of T cells using this novel SR was comparable to expansion kinetics using HS or FBS. It is important to point out that these T-cell expansions included both CD4^+^ and CD8^+^ T cells. These *ex vivo* expanded T cells were highly amenable for lentivirus-mediated gene transduction for potential application in the generation of gene-modified effector cells encoding either tumour-specific chimeric antigen receptors or T-cell receptors. Interestingly, we observed a significantly improved expansion of CD8^+^ T cells directed towards subdominant viral antigens from EBV in growth medium supplemented with CTS Immune Cell SR. Furthermore, phenotypic profiling showed that the EBV-specific effector cells expanded with CTS Immune Cell SR displayed a higher proportion of CD28^+^, CD62L^+^ and CD27^+^ T cells (central memory) when compared with the cells expanded with FBS. These observations are indeed important as the *in vivo* proliferative capacity and long-term survival of central memory T cells is enhanced when compared with terminally differentiated T cells when used for adoptive immunotherapy.^32, 33^ It is important to note that in spite of some phenotypic differences, antigen-specific T cells expanded with CTS Immune Cell SR displayed a polyfunctional profile comparable to the T cells expanded with FBS. Most of these T cells expressed CD107, TNF, IFN-γ and IL-2 following stimulation with viral peptide epitopes.

Earlier studies based on both human and murine models have shown that different γC-cytokines (for example, IL-2, IL-7 and IL-15) can have a dramatic impact on *in vitro* expansion of antigen-specific T cells.^25, 26, 27, 34^ To further refine our T-cell expansion protocol using CTS Immune Cell SR, we stimulated human PBMC with viral antigens in combination with different γC-cytokines (IL-2, IL-7, IL-15 and IL-21). Consistent with previously published reports, IL-2 alone or in combination with IL-21, IL-7 and/or IL-15 showed strong proliferation of both CD8^+^ and CD4^+^ T cells. These T-cell expansions were comparable to those seen with growth medium supplemented with FBS in the absence of IL-2, the total yield was reduced. Taken together, the data presented in this study provide clear evidence that CTS Immune Cell SR is highly efficient in expanding both polyclonal and virus-specific T cells directed against both dominant and subdominant antigens. Furthermore the cost of manufacturing T-cell therapy using the CTS Immune Cell SR is comparable to traditional growth medium supplemented with serum. These observations are highly relevant for the potential clinical application of T-cell therapies for infectious complications in immunosuppressed transplant patients and also for cancer patients. It will be important in future studies to formally assess the potential application of CTS Immune Cell SR in clinical settings where the patients are severely immunocompromised or lymphopenic.

## Methods

### Activation of polyclonal T cells using CTS Dynabeads CD3/CD28

Polyclonal T cells from freshly isolated PBMC (from healthy blood donors) were isolated and activated *ex vivo* using CTS Dynabeads CD3/CD28 (Life Technologies) at a bead to T cell ratio of 3:1 and cultured for 2 weeks. T cells were seeded 1 × 10^6^ T cell ml^−1^ in cell culture media with 100 U ml^−1^ IL-2 (Life Technologies) in tissue culture wells. Media used were XVIVO 15 (Lonza, Walkersville, MD, USA), CTS OpTmizer T-cell Expansion SFM, or CTS AIM-V (Life Technologies). Pooled human AB serum (2 or 5% as indicated, Life Technologies) or CTS Immune Cell SR (percentage indicated in figure legends, Life Technologies) were added to the media for comparison. All cell culture media were supplemented to a final concentration of 6 mm glutamine (Life Technologies). Cell cultures were fed with fresh media every 1–3 days as needed. At the end of culture, cells were counted and phenotype was analysed using flow cytometer (CD4 and CD8, Life Technologies). Cell acquisition was performed using a BD LSRII (BD Biosciences, Franklin Lakes, NJ, USA). Post-acquisition analysis was performed using FACSDiva software (BD Biosciences).

### Lentiviral transduction of polyclonal T cells

After 16–20 h of stimulation with CTS Dynabeads CD3/CD28 (bead to T-cell ratio 3:1) as described above, 1 multiplicity of infection of pELNS-GFP lentiviral vector (kindly provided from Dr James Riley and Andrew Medvec at Department of Microbiology at University of Pennsylvania) was added to the cultures. Efficiency of transduction was assessed at day 7 by flow cytometry. Cell acquisition was performed using a BD LSRII (BD Biosciences). Post-acquisition analysis was performed using FACSDiva software (BD Biosciences).

### Activation of polyclonal T cells using OKT-3 monoclonal antibody, feeder cells and high dose IL-2

Polyclonal T cells from freshly isolated PBMC (from healthy blood donors) were isolated using Dynabeads Untouched T cells Kit (Life Technologies) and activated *ex vivo* using OKT3 (30 ng ml^−1^, eBioscience, San Diego, CA, USA), pooled irradiated feeder cells (1:100) and high dose IL-2 according to the rapid expansion protocol described by Rosenberg lab (Dudley *et al.*). Cells were seeded 1 × 10^5^ T cell ml^−1^, 5 × 10^7^ irradiated feeder cells ml^−1^ in cell culture media with 3000 U ml^−1^ IL-2 (Life Technologies) in tissue culture wells and grown for 2 weeks. Cell cultures were fed with fresh media every 1–3 days as needed. Media used were XVIVO-15 (Lonza), CTS OpTmizer T cell Expansion SFM, or AIM-V CTS (Life Technologies). Pooled human AB serum (2 or 5% as indicated) or 10% CTS Immune Cell SR were added to the media for comparison. All cell culture media were supplemented to a final concentration of 6 mm glutamine (Life Technologies). At the end of culture, cells were counted and phenotype was analysed using flow cytometer (CD4, CD8 and CD62L).

### Activation of CMV-specific T cells

CMV-specific T cells were generated using a pool of CMV-specific CD8^+^ T-cell epitopes (JPT Peptide Technologies GmbH, Berlin, Germany) as outlined previously.^18^ Briefly, 2.7 × 10^6^ PBMC were co-cultured in a 24-well plate with peptide pulsed autologous PBMC at a responder to stimulator ratio of 2:1, in either RPMI–10% FBS or in OpTmizer supplemented with 5% SR. On day 3, and every 3–4 days thereafter, the cultures were supplemented with the appropriate growth medium containing 120 IU ml^−1^ recombinant IL-2. On day 14, cells were harvested, cell number and viability determined using trypan blue exclusion, specific T cells using a standard IFN-γ intracellular cytokine assay as described previously.^18^

### Activation of EBV-specific T cells

LMP/EBNA1-specific T cells were generated using the AdE1-LMPpoly adenoviral vector as outlined previously.^21^ Briefly, 2.7 × 10^6^ PBMC were co-cultured in a 24-well plate with autologous PBMC infected with AdE1-LMPpoly (multiplicity of infection of 10:1) at a responder to stimulator ratio of 2:1, in either RPMI-1640 supplemented with 10% FBS, or in CTS OpTmizer T-cell Expansion SFM (OpTmizer) supplemented with 5 or 10% CTS Immune Cell SR or 10% Human AB serum. On day 3, and every 3–4 days thereafter, the cultures were supplemented with the appropriate growth medium containing 120 IU ml^−1^ recombinant IL-2. On day 14, cells were harvested, cell number and viability were determined using trypan blue exclusion, then tested for LMP- and EBNA1-specific T cells using a standard IFN-γ intracellular cytokine assay as described previously.^21^

### Polyfunctional cytokine analysis

T cells were assessed for polyfunctional cytokine production (IFN-γ, TNF and IL-2) and degranulation (CD107a), as described previously.^18^ Briefly, T cells were stimulated with cognate peptide and incubated for 4 h in the presence of FITC anti-CD107a, GolgiPlug and GolgiStop (BD Biosciences). Cells were then incubated with PerCP-Cy5.5 anti-CD8 and PE-Cy7 anti-CD4, fixed and permeabilised, then incubated with AF700 anti-IFN-γ, APC anti-TNF and PE anti-IL-2. Cell acquisition was performed using a BD LSR Fortessa (BD Biosciences). Post-acquisition analysis was performed using FlowJo software (TreeStar, Ashland, OR, USA).

### Phenotypic analysis of cultured T cells

*In vitro* expanded T cells were incubated for 20 min with the APC labelled HLA B35/HPVGEADYFEY pentamer (ProImmune, Oxford, UK), then incubated for a further 30 min at 4 °C with V500 anti-CD8, PE-Cy7 anti-CD4, PE anti-CD27, PerCP-Cy5.5 anti-CD28, FITC anti-CD62L and biotin anti-CD57; followed by incubation with BV421 streptavidin. To assess the expression of Perforin and Granzymes B and K, T cells were incubated with the APC labelled HLA B35/HPVGEADYFEY pentamer, incubated with PerCP-Cy5.5 anti-CD8, then fixed and permeabilised using the BD transcription factor buffer kit. Cells were then incubated with BV421 anti-perforin (eBioscience), Alexa Fluor 700 anti-Granzyme B (BD Biosciences) and FITC anti-Granzyme K (Santa Cruz Biotechnology, Dallas, TX, USA). All cell acquisition was performed using a BD LSR Fortessa. Post-acquisition analysis was performed using FlowJo software.

### Statistical analysis

All statistical analysis was performed using GraphPad Prism software (GraphPad, La Jolla, CA, USA). Differences were considered to be statistically significant where *P*<0.05.

## Notes

GO, TA and KWS are employees of Thermo Fischer Scientific, the manufacturers of the novel serum replacement supplement outlined in this publication. The remaining authors declare no conflict of interest.
